# Boosting Vaccine Response in Autoimmune Rheumatic Disease Patients With Inadequate Seroconversion: An Analysis of the Immunogenicity of Vector-Based and Inactivated Vaccines

**DOI:** 10.7759/cureus.55764

**Published:** 2024-03-07

**Authors:** Anuroopa Vijayan, Aswathy Sukumaran, Sara Jones, Aby Paul, Sakir Ahmed, Pankti Mehta, Manju Mohanan, Santhosh Kumar, Sreekumar Easwaran, Padmanabha Shenoy

**Affiliations:** 1 Rheumatology, Dr Shenoys CARE, Kochi, IND; 2 Rheumatology, Sree Sudheendra Medical Mission, Kochi, IND; 3 Pharmacy, Dr Shenoys CARE, Kochi, IND; 4 Pathogen Biology, Rajiv Gandhi Centre for Biotechnology, Thiruvananthapuram, IND; 5 Rheumatology, Kalinga Institute of Medical Sciences, Bhubaneswar, IND; 6 Clinical Immunology and Rheumatology, King George's Medical University, Lucknow, IND; 7 Microbiology, Dr Shenoys CARE, Kochi, IND; 8 Cancer Biology, Rajiv Gandhi Centre for Biotechnology, Thiruvananthapuram, IND; 9 Virology, Institute for Advanced Virology, Thiruvananthapuram, IND; 10 Clinical Immunology and Rheumatology, Dr Shenoy's CARE, Kochi, IND

**Keywords:** heterologous vaccine, immunogenicity, immunocompromised patient, booster vaccine, covid 19 vaccine

## Abstract

Background: An additional dose of COVID-19 vaccine is being offered to vaccinated people, especially those immunocompromised. The most widely available vaccines in India are the adenoviral vector-based AZD1222 (ChAdOx1 nCoV-19) and the heat-inactivated (BBV152). This study investigated the efficacy of both vaccines in patients with autoimmune rheumatic diseases (AIRD).

Objectives: To compare final anti-SARS-CoV-2 antibody titers, neutralization of pseudovirions by these antibodies, and T cell responses between patients of AIRD who had received the third dose of AZD1222 and BBV152 vaccines.

Methods: Patients with stable AIRD who had completed two doses of COVID-19 vaccination but had a suboptimal response (anti-receptor binding domain (RBD) antibody<212) were randomized (1:1) to receive either AZD1222 or BBV152 as a booster dose. Patients with previous hybrid immunity or those who developed COVID-19 during the trial were excluded. Antibody titers, neutralization of Wuhan and Omicron pseudovirions, and interferon release by T cells (enzyme-linked immunosorbent spot (ELISpot)) in response to the Spike antigen were measured four weeks after this booster dose.

Results: 146 were screened, 91 were randomized, and 67 were analyzed per protocol. The third dose improved antibody titers (p<0.001), neutralization of the Wuhan strain (p<0.001), and T cell interferon release (p<0.001) but not neutralization of the Omicron strain (p=0.24). Antibody titers were higher (p<0.005) after ADZ1222 boost (2,414 IU (interquartile range (IQR): 330-10,315)) than BBV1222 (347.7 IU (0.4-973)). Neutralization of the Wuhan stain was better (AZD1222: 76.6%(23.0-95.45) versus BBV152 (32.7% (0-78.9), p=0.03 by ANCOVA). Neutralization of Omicron (0 (0-28.4) vs 0 (0-4.8)) and T cell interferon release (57.0 IU (23.5-95) vs 50.5 IU (13.2-139)) were similar.

Conclusion: The third dose improved all parameters of immunogenicity in AIRD patients with previous inadequate responses except Omicron neutralization. The vector-based vaccine exhibits notable efficacy, particularly in antibody titers and neutralizing the Wuhan strain.

Trial registration: CTRI/2021/12/038928

## Introduction

The most effective way to combat the SARS-CoV-2 pandemic is a population-wide vaccination strategy. Immune responses to vaccines vary across individuals and this is especially relevant when it comes to immunocompromised patients. They are at high risk for infection, can have worse outcomes, and may harbor the virus for prolonged periods with a predisposition for the development of viral mutations resulting in breakthrough epidemics with emerging strains [1-3].

A meta-analysis reported that the rate of seroconversion for COVID-19 vaccination in immunocompromised individuals is approximately half of immunocompetent controls. Furthermore, the antibody titers obtained are lower and tend to wane off faster in immunocompromised patients [4,5]. As the development of humoral response is now regarded as a suitable analog for protection against breakthrough infections, its absence is frequently interpreted as a warning sign of insufficient vaccination response [6]. To overcome this, an additional vaccine dose is recommended in various countries for people on immunosuppressants or with diseases compromising their immune systems [4].

Many studies have supported a boosting of immunity in immunosuppressed patients as well as induction of humoral immunity in those with absent responses after an additional vaccine dose [7]. The World Health Organization and Centre for Disease Control both recommended a booster dose in immunocompromised individuals [8,9]. However, most of these studies were done in cancer patients and transplant recipients. There is limited data on the effect of booster dose vaccine in patients with autoimmune rheumatic diseases (AIRDs), which have both a dysregulated immunity and immunosuppressant medication use [10]. In a majority of the studies, humoral responses have been studied without T cell responses and the preponderant vaccines used were mRNA vaccines. Similarly, another pertinent question is regarding the benefits of heterologous versus homologous vaccination, especially for the combination of viral vector versus inactivated whole virion vaccines [10,11].

In India, the most widely available vaccines are the adenoviral vector-based AZD1222 (ChAdOx1 nCoV-19) and the inactivated (BBV152). In this study, we aimed to investigate the efficacy of these two vaccines given in various combinations across the primary and booster vaccinations in patients with AIRDs.

## Materials and methods

Objective and design

It’s a single-center, open-labeled, randomized controlled trial that compared the effects of a third (or booster) dose of either AZD1222 or BBV152 in patients with AIRDs with stable disease activity and had completed two doses of COVID-19 vaccination with suboptimal response. A suboptimal response was defined as an antibody titer of less than 212 IU/mL at four weeks after the second vaccine dose [12].

Inclusion criteria

Patients with AIRDs who have completed vaccination with either two doses of AZD1222 and BBV152 vaccines were included if they were above 18 years of age; were being treated for a diagnosed AIRD; on stable doses of immunosuppressant for more than three months and stable doses of more than three weeks in case of corticosteroids; and who remained seronegative or developed a suboptimal response (antibody titers <212 IU/mL) after two doses of COVID-19 vaccination. The cut-off of 212 IU/mL was obtained from our previous study that had shown correlation of higher levels of antibody with the neutralization of virion particles [13].

Exclusion criteria

Patients with unstable disease activity and a history of flare following vaccinations; those who had ever received rituximab in the past and had non-repopulated peripheral B cell counts; with a history of COVID-19 infection; and those with a history of allergic reaction to the vaccine or its components were excluded. If patients developed COVID-19 during the trial, they were also excluded.

Allocation

**Figure 1:**
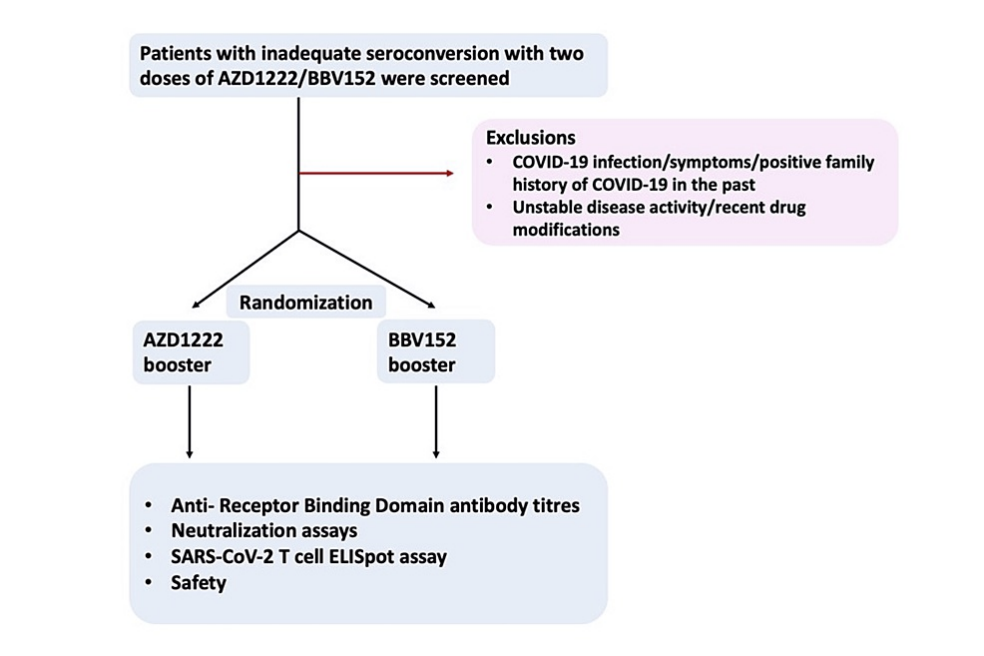
Study flow chart

After written informed consent, participants were randomized (1:1) to receive either AZD1222 or BBV152 as a booster dose. Block randomization was used to ensure that there were equal participants in both groups. Patients were followed up every two weeks for three months telephonically to determine side effects, disease flares, and COVID-19-related symptoms/proven COVID-19 infection if any (Figure 1).

Outcomes

Outcomes were anti-receptor binding domain (RBD) antibody titers, neutralization of SARS-COV-2 pseudo virion particles, and T cell responses to the SARS-CoV-2 Spike protein. The secondary objective included the study of the short-term safety of booster vaccination up to seven days post-vaccination.

Laboratory tests

Peripheral blood was collected from all participants at four weeks after the booster dose. Anti-RBD antibody titers against the SARS-CoV-2 were measured using The Elecsys® Anti-SARS-CoV-2 S immunoassay (Roche Diagnostics International Ltd, Switzerland).

The SARS-CoV-2 surrogate virus neutralization test (sVNT) kits (GenScript) directed against the original Wuhan strain and the Omicron strain were used to estimate neutralizing antibodies.

Enzyme-linked immunosorbent spot (ELISpot) assay was used to measure interferon-gamma (IFN-Ɣ) release using Mabtech ELISpot Plus: Human IFN-Ɣ(ALP) kit by T cell responses to peptide pool spanning the spike antigen of the SARS-CoV-2 virus (Mabtech, Sweden).

T cell response

Peripheral blood mononuclear cells (PBMCs) were isolated from a whole blood sample collected in lithium heparin tubes (BD bioscience) within eight hours using lymphoprepTM (Stem Cell Technologies) density gradient centrifugation in a Sep Mate tube (Stem Cell Technologies). The cells were diluted 1:1 with 1X phosphate-buffered saline (PBS; Himedia), pH 7.4. The cells were washed twice by the addition of 1X PBS with 2% heat-inactivated fetal bovine serum (FBS) (Sigma-Aldrich) and centrifugation. The pellet was re-suspended in Roswell Park Memorial Institute (RPMI) medium (RPMI 1640 medium; Gibco) supplemented with 10% heat-inactivated FBS, 2mM/liter glutamine, 100 IU/ml penicillin, 100g/ml streptomycin, 1mM/liter sodium pyruvate. The PBMCs thus obtained were diluted in a complete RPMI medium to a final concentration of 250000 cells/ mL. The SARS-CoV-2 T cell ELISpot assay was performed using pre-coated 96 well plates (mAB 1-D1K; Mabtech, Nacka Strand, Sweden). Plates were washed four times with filtered PBS (Gibco) and blocked with a complete RPMI (cRPMI) medium containing 10% batch-tested FBS (Sigma-Aldrich). For each subject, 250,000 PBMCs per well were stimulated for 48 hours with SARS-CoV-2 S-defined peptide pools containing 100 peptides from human SARS-CoV-2 virus (2µg/ml) (Mabtech), mAB CD3-2 (1:1,000 dilution) (Mabtech) as positive control and cRPMI with cells as negative control. The numbers of IFN-γ spot-forming cells (sfc) per 250,000 cells per ml were counted using the Autoimmun Diagnostika GmbH ELISPOT Reader system and analysed using Autoimmun Diagnostika GmbH ELISpot software, version 7.0. Mean spot counts for negative control wells were subtracted from the mean of test wells to generate normalized readings, and are represented as IFN-γ sfc) per quarter million PBMCs. The value of (median + 2 × SD) was used as the lower limit to indicate a positive response in the test samples and was measured as 40 spots per million PBMCs.

Sample size estimation

A convenient sample was included after screening 146 patients to have an approximately equal number of participants who had completed AZD1222 or BVV152 vaccination. A per-protocol analysis was planned after excluding any patients who developed COVID-19 at any point during the trial period.

Statistical analysis

All the data collected were coded and entered in a Microsoft Excel spreadsheet in duplicate to avoid errors. Analysis and graphical representation were made on R (version 4.2). The normality of data was checked by the Shapiro-Wilk test and described as mean (SD) or median (interquartile range) depending on the normality of data for quantitative variables and as frequency/percentage for categorical variables. Kruskal Wallis test was used for comparing continuous variables across groups. Pearson Chi-square test was used for comparing categorical variables across groups. Wilcoxon signed ranks test was used for paired analysis. A p-value of <0.05 was deemed as statistically significant.

Sub-group analyses were carried out to determine the difference between homologous and heterologous vaccination strategies and between the use of the two vaccines as boosters. All these analyses were carried out using ANCOVA (analysis of co-variance) controlling for the baseline (post-second vaccine dose) values.

Ethics approval

Ethics approval was obtained from the Sree Sudheendra Medical Mission and the trial was registered at the CTRI site (No CTRI/2021/12/038928). Written informed consent was obtained from each participant at the time of enrollment.

## Results

**Figure 2:**
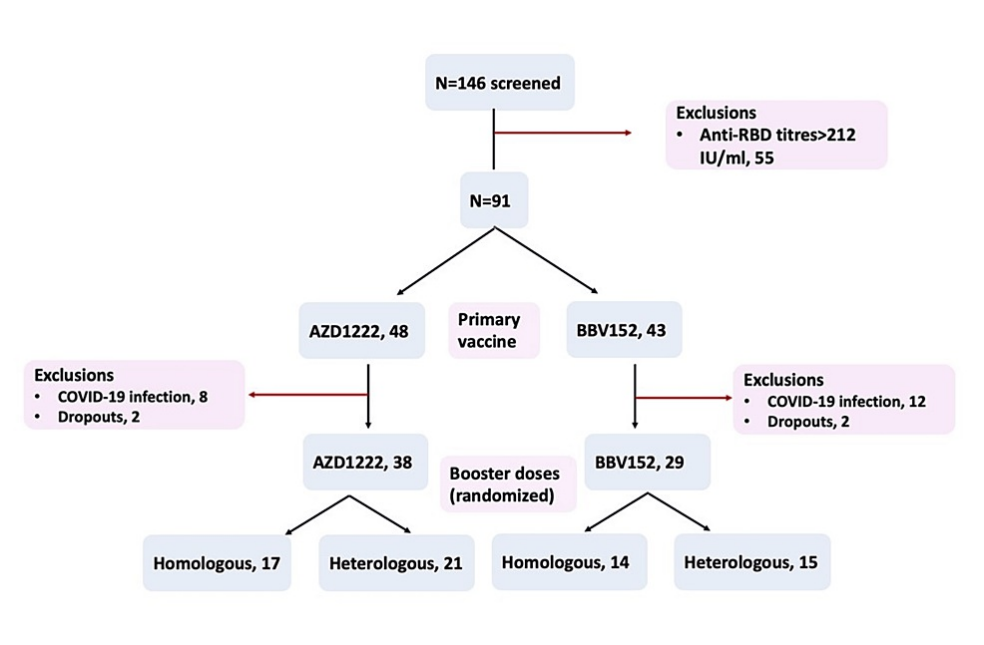
Randomization of the study participants

146 patients with AIRD with a complete primary immunization with two doses of either AZD1222 or BBV152 were screened, and 55 of them were excluded as they had a good antibody response (>212 IU/mL). The remaining 91 were randomized to AZD1222 and BBV152 booster groups of which 38 and 29 completed the study, respectively, in the two groups after excluding COVID-19 infections and dropouts. Thus, 67 patients were analysed by a per-protocol analysis (Figure 2).

Demographics

The baseline demographic characteristics of the study participants are depicted in Table 1.

**Table 1:** Baseline demographics IQR: Interquartile range; CTDs: Connective tissue disease; MMF: Mycophenolate mofetil

Demographics		Median (IQR)/No (%)
Age		55 (48.5-62)
Gender	Male	10 (14.9%)
	Female	57 (85.1%)
Primary vaccine	AZD1222	38 (56.7%)
	BBV152	29 (43.3%)
Primary vaccine + Booster vaccine	AZD1222 homologous	17 (25.4%)
	AZD1222 with BBV152 booster	21 (31.3%)
	BBV152 with AZD1222 booster	15 (22.4%)
	BBV152 homologous	14 (20.9%)
Disease	Rheumatoid arthritis	58 (86.56%)
	Spondyloarthritis	2 (2.98%)
	Lupus	1 (1.49%)
	Systemic sclerosis	1 (1.49%)
	Other CTDs	5 (7.46%)
Drugs	Hydroxychloroquine	38 (56.71%)
	Sulfasalazine	8 (11.94%)
	Methotrexate	33 (49.25%)
	Tofacitinib	11 (16.41%)
	Leflunomide	4 (5.97%)
	MMF	7 (10.44%)
	Tacrolimus	1 (1.49%)
	Azathioprine	2 (2.98%)
	Rituximab	2 (2.98%)

The age of the enrolled participants for the booster dose was 55 (IQR: 48.5-62) years. The most common diagnosis was rheumatoid arthritis (58, 74.6%) while the most commonly used disease-modifying anti-rheumatic drugs (DMARDs )were hydroxychloroquine 28% (56.7) and methotrexate in 33% (49.3).

Immune response after the third dose

**Figure 3:**
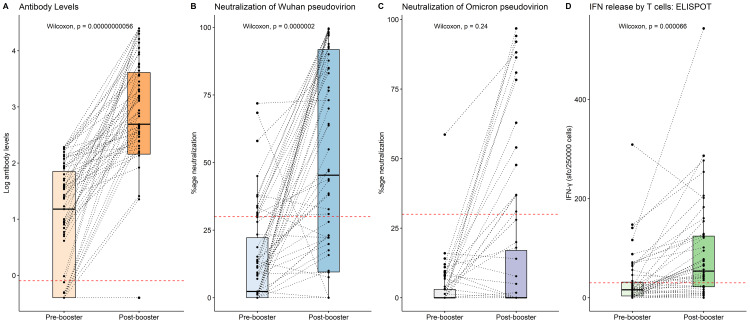
Immune response after third dose vaccine in terms of (A) antibody levels, (B) Wuhan pseudovirion neutralization, (C) Omicron pseudovirion neutralization, (D) IFN release Bar diagrams showing the immune response after third dose vaccine. Wilcoxon signed ranks test was used for comparison. p value < 0.05 was statistically significant.

Overall, the antibody titers improved from median 15.14 IU/mL (IQR: 0.4-71.6 IU/mL) at baseline to 491.6 IU/mL (144-4,107 IU/mL) at four weeks after the third dose (p<0.001) (Figure 3A). Neutralization of the Wuhan strain pseudovirus was significantly higher after the booster dose (from median 2.3% (0-22.2) to 45.32% (9.5-91.8); p< 0.001), but Omicron neutralization did not show a significant difference (from median 0% (0-3) to 0% (0-17); p 0.24) (Figure 3B, 3C). Even after the third dose, none of the sera had the recommended neutralization of at least 30% against the Omicron pseudovirus. IFN release by T cells was significantly higher after the booster dose (Figure 3D) (16 (3.5-31.5) IFN-γ sfc/250,000 cells to 53.8 (22-12) IFN-γ sfc/250,000 cells; p<0.0001).

Heterologous versus homologous booster

Subgroup analysis was carried out to compare homologous boosting (same vaccine to boost) versus heterologous boosting (boosting by the other vaccine). There was no difference in antibody levels (heterologous: 684 IU/mL (172-4,774 IU/mL) vs homologous: 462 IU/mL (108-2,518 IU/mL); p> 0.05) or in the neutralization of the Wuhan pseudovirus (heterologous: 55.3 (10.1-91.6)% vs homologous: 40.9 (9.84-90.6); p>0.05) or the Omicron pseudovirus (heterologous: 0 (0-28.2)% vs Homologous: 0 (0-1.25); p>0.05). However, it may be noted that in homologous booster, there is practically no response against the omicron pseudovirus.

In the T cell interferon release assay, heterologous boosting was numerically better (heterologous: 57.5 (37.1-114.0) IFN-γ sfc/250,000 cells vs homologous: 47.8 (13.5-128.0) IFN-γ sfc/250,000 cells; p=0.06 in ANCOVA controlled for baseline ELISpot values) though it stopped just short of reaching statistical significance.

AZD1222 versus BBV152 booster

**Figure 4:**
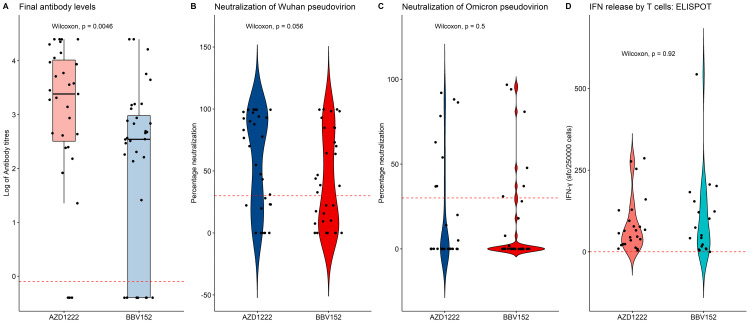
Comparison of immune response: AZD 1222 versus BBV 152 booster dose Bar diagram and violin plots showing antibody response, Wuhan and omicron pseudovirion neutralization, IFN release, comparison using Wilcoxon signed ranks test

As compared to BBV152, the use of AZD1222 as the booster irrespective of the primary immunization led to better B cell responses (Figure 4).

**Figure 5:**
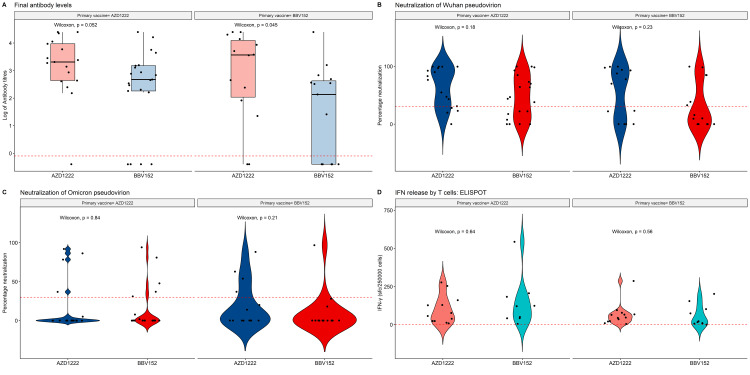
Effects of booster vaccine in terms of antibody levels, Wuhan and Omicron neutralization, IFN release stratified by the primary vaccine Bar diagrams and violin plots show the immune response.

The total antibody response (ADZ1222: 2,414 IU/mL (330-10315) vs BBV152: 348 IU/mL (04-973); p= 0.026) and the Wuhan neutralizing capacity (ADZ1222: 76.6%(23-95.4) vs BBV152: 32.7%(0-78.9); p= 0.026] were better boosted with the AZD1222 vaccine while Omicron neutralization [ADZ1222: 0%(0-28.4) vs BBV152: 0%(0-4.8); p=0.9) and T cell responses (ADZ1222: 57 (23.5-95) IFN-γ sfc/250,000 cells vs BBV152: 50.5% (13.2-139) IFN-γ sfc/250,000 cells; p=0.6) were statistically same. The effects of the boosters stratified by the primary vaccine used are presented in Figure 5.

BBV152 homologous versus other combinations

Persons who had received three doses of BBV152 were compared against all other combinations (2 BBV152 + 1 ADZ1222 / 2 ADZ1222 + 1 BBV152 / 3 ADZ1222). The seroconversion was achieved only in 54% (7/13) in the BBV152 homologous group as compared to 83.3% (45/54) in the rest (p=0.022; χ2 test). The BBV152 homologous group had lower antibody levels (median 136 (0.4-429.25) vs 1077 (232.25-5726.5); p=0.007) and lower neutralization of the Wuhan pseudovirion (10 (0-61.68) vs 63.7 (22.2-92.9); p =0.047). However, Omicron neutralization (0 (0-0) vs 0 (0-25.5); p=0.3) and T cell interferon release 19.5 (8.9-94.6) IFN-γ sfc/250,000 cells vs 60.75 (26.5-126.1) IFN-γ sfc/250,000 cells; p=0.15) were similar.

Safety and adverse effects

The most common systemic adverse effects were fever (4; 5.97%) and disease flare (3; 4.4%). There was no significant differences across the four groups except for higher fatigue in the BBV152 heterologous group (Table 2).

**Table 2:** Adverse events reported after the booster dose Adverse events after the booster dose expressed as N (%), p value obtained using Pearson Chi-square test. p value < 0.05 was statistically significant.

	Total (n=67)	AZD1222 homologous	AZD1222 heterologous	BBV152 homologous	BBV152 heterologous	P value
Swelling at site	1 (1.4)	0 (0)	1 (5)	0 (0)	0 (0)	0.525
Fever	4 (5.97)	0 (0)	2 (10)	0 (0)	2 (15.4)	0.222
Fatigue	2 (2.9)	0 (0)	0 (0)	0 (0)	2 (15.4)	0.044*
Chills	1 (1.4)	0 (0)	0 (0)	0 (0)	1 (7.7)	0.263
Myalgia	1 (1.4)	0 (0)	0 (0)	0 (0)	1 (7.7)	0.263
Rash	1 (1.4)	0 (0)	1 (5)	0 (0)	0 (0)	0.535
Disease flare	3 (4.4)	0 (0)	2 (10)	0 (0)	1 (8.3)	0.377

## Discussion

The effect of an additional third dose of a COVID-19 vaccine was studied in patients with AIRD with an inadequate response to primary vaccination. Outcomes were measured with anti-RBD antibody titers, neutralization of Wuhan and Omicron pseudo virions, and T cell IFN-Ɣ response after stimulation with SARS-CoV-2 specific S-protein. All these parameters were boosted by the third dose except for Omicron neutralization. Both the booster vaccines had comparable adverse effects and no serious adverse effect was noted after the third dose. The use of AZD1222 as the booster led to better antibody titers and improved neutralization of the Wuhan strain. Patients who had received three doses of BBV152 had the least seroconversion with the lowest medians antibodies titers.

Epidemiological studies have already shown the effectiveness of the booster dose in reducing the risks for breakthrough infections, regardless of the type of rheumatic disease or immunosuppressant medications [14]. Some studies have looked at humoral responses to booster doses in immunocompromised individuals. Two cohort studies from France analysed homologous mRNA vaccine boosters in solid organ transplant recipients and found an increase in humoral response rates from 40-50% after primary vaccination to 68-69% after the booster dose [15,16]. In a subset with inadequate response to primary immunization, the response after boosters varied from 6% to 88% across various studies. However, these studies have severely immunocompromised patients and hence may not be representative of patients with AIRD [7].

Two recent trials have studied the effect of boosters in patients with AIRD. One of them studied the effect of either an mRNA or viral vector vaccine booster following primary immunization with mRNA vaccines in patients on rituximab who had not seroconverted after primary immunization [10]. Overall, 27% of patients seroconverted after the booster dose and there was no difference in the homologous and heterologous groups statistically, but a trend towards lower rates of seroconversion was observed in the viral vector vaccine group. T cell responses were better in the viral vector group than mRNA group (100% versus 83%) [10]. Another trial included patients with malignancies, solid organ transplants, and AIRD who failed to seroconvert after primary mRNA vaccination and assessed response to viral vector or mRNA vaccine boosters. The rate of seroconversion and median antibody titers were significantly higher in the mRNA booster group without a significant difference in the T cell responses [11]. Current guidelines also mention the third dose in patients with AIRD [17]. What was novel in our study is that we had focused only on those who had a previous inadequate response to two doses of the primary vaccination. We were able to show that the booster is efficacious even in such a population.

We did not find a statistically significant difference in the antibody responses across the homologous and heterologous groups. Data from booster doses in healthy individuals have shown mixed responses with homologous or heterologous boosters. A study from the United States had shown a significant increase in antibody titers, neutralization potential, and T cell response with mRNA vaccine boosters. The increase was the lowest in the Ad26.COV2-S booster group irrespective of the primary immunization [18]. Another study from Brazil that included patients who had received the CoronoVac vaccine for primary immunization, found significantly better antibody responses in the heterologous booster group (Ad26.COV2-S, BNT162b2, ChAdOx1 nCoV-19) compared to the homologous group. It found the viral neutralization to be also significantly higher in the heterologous group (100% versus 83%, p<0.0001) [19]. A population-based study in Malaysia analysed immunization by BNT162b2 and by CoronaVac in various combinations and found no difference between heterologous and homologous immunization. The only exception was in those with age more than 60 years in whom heterologous vaccination induced better protection against breakthrough infections [20]. Another study has reported synergistic effects of the ChAdOx1 nCoV-19 vaccine when used heterologously with mRNA vaccines [21]. These varied results in different studies are due to the differences in the type of vaccines, definitions of inclusions as well as sample sizes. Possibly the superior performance of heterologous vaccines is limited to specific vaccine combinations.

This study has clearly shown that inactivated whole virion vaccines have demonstrated less efficacy compared to alternative vaccine combinations. This is in line with our previous work and similar findings have been seen in a meta-analysis [12,22]. Not only antibody titers were low, but seroconversion did not occur in around half of the cohort who had three doses of BBV152. Though the difference in the T cell stimulation assay was not statistically significant, this too was numerically smaller and possibly statistical significance was not reached due to a type II error. This provides an important message that patients who had received two doses of an inactivated vaccine previously should be given a booster other than the heat-inactivated vaccine. The safety of the booster dose in patients with AIRD has already been established [23] and we did not find any additional danger signals.

The distinct strengths of our study include randomization before vaccination to minimize the effect of confounders, assay of immune parameters at a fixed interval after the vaccination, inclusion of both B cell and T cell data, and assessment of neutralization of both the classical Wuhan and the evolved Omicron strain. Limitations include attrition of the sample due to many patients developing breakthrough COVID-19 infections before the completion of the trial. Furthermore, a longer follow-up will be required to assess the risk of breakthrough infections and hospitalizations for the real-life effectiveness of the boosters.

## Conclusions

The booster dose administered, whether vector-based or inactivated vaccine, effectively enhanced various parameters of immunogenicity in AIRD patients exhibiting inadequate responses to initial vaccination. However, notable exceptions were observed in Omicron neutralization. The trial did not highlight any distinct advantage in employing a heterologous booster, except in cases where both primary doses comprised inactivated vaccines. While demonstrating promising outcomes, the vector-based vaccine notably exhibited higher antibody titers and better neutralization against the Wuhan strain. Further efforts should focus on developing strategies to specifically target Omicron neutralization. Patients who had received two doses of inactivated vaccines previously should be boosted by a (heterologous) vaccine working on another principle. Furthermore, the administration of the third dose did not yield significant systemic or local adverse effects.
